# Analysis of the Chemical, Antioxidant, and Anti-Inflammatory Properties of Pink Pepper (*Schinus molle* L.)

**DOI:** 10.3390/antiox10071062

**Published:** 2021-06-30

**Authors:** Min Jeong Kim, Dae Won Kim, Ju Gyeong Kim, Youngjae Shin, Sung Keun Jung, Young-Jun Kim

**Affiliations:** 1 School of Food Science and Biotechnology, Kyungpook National University, Daegu 41566, Korea; excellent8@knu.ac.kr (M.J.K.); jgrla23@gmail.com (J.G.K.); 2 Department of Food Science and Technology, Seoul National University of Science and Technology, Seoul 01811, Korea; kdw3566@seoultech.ac.kr; 3 Department of Food Engineering, Dankook University, Cheonan, Chungnam 31116, Korea; ys234@dankook.ac.kr; 4 Institute of Agricultural Science & Technology, Kyungpook National University, Daegu 41566, Korea

**Keywords:** nutraceutical, compound compositions, reactive oxygen species, mitogen-activated protein kinase, nuclear factor kappa-light-chain-enhancer of activated B cells

## Abstract

Here, we compared the chemical properties and antioxidant effects of black pepper (*Piper nigrum* L.) and pink pepper (*Schinus molle* L.). Additionally, the antioxidant and anti-inflammatory capacities of pink pepper were measured to determine nutraceutical potential. Pink peppers from Brazil (PPB), India (PPI), and Sri Lanka (PPS) had higher Hunter a* (redness) values and lower L* (lightness) and b* (yellowness) values than black pepper from Vietnam (BPV). Fructose and glucose were detected in PPB, PPI, and PPS, but not in BPV. PPB, PPI, and PPS had greater 2,2-diphenyl-1-picrylhydrazyl and 3-ethylbenzothiazoline-6-sulphonic acid radical scavenging stabilities and higher total phenolic contents than BPV. BPV had higher levels of piperine than the pink peppers. Gallic acid, protocatechuic acid, epicatechin, and p-coumaric acid were detected only in the three pink peppers. PPB significantly suppressed lipopolysaccharide-induced reactive oxygen species production with increased Nrf2 translocation from cytosol to nucleus and heme oxygenase-1 expression. PPB and PPS significantly suppressed lipopolysaccharide-induced nitrite production and nitric oxide synthase expression by suppressing phosphorylation of p38 without affecting cell viability. Additionally, PPB and PPS significantly suppressed ultraviolet B-induced cyclooxygenase-2 expression by affecting the phosphorylation of ERK1/2 without cell cytotoxicity. These results suggest that pink pepper is a potential nutraceutical against oxidative and inflammatory stress.

## 1. Introduction

Reactive oxygen species (ROS), reactive molecules and free radicals derived from molecular oxygen, are responsible for the elimination of microbial invasion [1]. Although phagocytes, such as macrophages, are innate immune cells that are important for the elimination of pathogens, including viruses, bacteria, and parasites, the abnormal production of ROS causes oxidative stress and consequent cell death. Additionally, inflammation is closely related to ROS levels because unregulated immune response to a pathogen results in overproduction of ROS, which causes damage to the host organ [2]. Excessive production of nitric oxide (NO) also plays a critical role in inflammation. Thus, continuously high ROS and NO levels lead to chronic inflammation, which can result in the onset of various diseases, including atherosclerosis, rheumatoid arthritis, diabetes, and cancer [3,4]. Therefore, detoxification of ROS is a key step preventing inflammation and the subsequent development of chronic inflammatory diseases.

Heme oxygenase-1 (HO-1) is an Nrf2-regulated gene that has important antioxidant, anti-inflammatory, and antiapoptotic effects [5]. While Nrf2/HO-1 signaling plays a protective role in the regulation of oxidative stress and inflammation, NF-κB, a heterodimer of p65 and p50/p105, plays a central role in ROS- and pathogen-mediated inflammatory signaling cascades. In addition, mitogen-activated protein kinases (MAPKs) are involved in inflammation [6]. A previous study reported that ultrasonicated seaweed extract suppressed lipopolysaccharide (LPS)-induced nitric oxide synthase (iNOS) expression and nitrite production via regulation of MAPK phosphorylation without affecting the NF-κB signaling cascade in RAW264.7 cells [7]. Additionally, the phosphorylation of MAPKs is central in skin inflammation response to ultraviolet (UV) light exposure in vitro and in vivo [8,9]. Therefore, in addition to NF-kB, MAPKs may be a prime target for anti-inflammatory nutraceuticals.

*Schinus molle* L., also known as Peruvian pepper, Brazilian pepper, American pepper, Californian pepper, or molle de Peru, is a fast-growing evergreen tree [10]. Pink pepper is classified into the Californian/Peruvian type (*S. molle*) and the Brazilian type (*S. terebinthifolius*) [11]. Although it is unrelated to the black pepper (*Piper nigrum*), the pink fruits of *S. molle* are known as pink peppercorns and are used as alternatives to black pepper due to their flavor and pungency [12]. Research on pink pepper has primarily focused on the chemical composition and physicochemical characteristics of its essential oil and its antimicrobial activities [13,14]. The results of recent studies have revealed that polysaccharides from *S. molle* fruit have in vitro antioxidant, antigenotoxic, antidiabetic, and antihemolytic effects, as well as in vivo anti-inflammatory and antinociceptive properties [15]. However, the differences in the chemical compositions and the antioxidant and anti-inflammatory effects of *S. molle* fruits from different countries remain unclear.

In the present study, we aimed to compare the chemical and biological activities of commercially available pink peppers from Brazil (PPB), India (PPI), and Sri Lanka (PPS) and black pepper from Vietnam (BPV) as a health functional food ingredient. The three pink peppers were sorted based on chromaticity analysis and photographs and were found to have higher redness than black pepper. Previous studies have focused only on high piperine content and NF-kB pathway of black pepper; however, despite the low piperine content of pink pepper, the total flavonoid content and radical scavenging activity of pink pepper were higher than those of black pepper, and, hence, we focused on the effects of pink pepper on ROS production, HO-1, and Nrf2 translocation in RAW264.7 cells and ultraviolet B (UVB)-induced skin inflammation in HaCaT cells.

## 2. Materials and Methods

### 2.1. Materials

Pink peppers (*S. molle* L.) gathered in Brazil, India, and Sri Lanka and black peppers (*Pieper nigrum* L.) gathered in Vietnam were purchased from the local marker. Antibodies against HO-1, iNOS, COX-2, p-p65 (Ser536), p65, p-p38, p38, p-JNK, JNK, p-ERK, and ERK were purchased from Cell Signaling Technologies (Danvers, MA, USA). Additionally, β-actin was obtained from Santa Cruz Biotechnology, Inc. (Santa Cruz, CA, USA), and 2′,7′-dichlorofluorescein diacetate and lipopolysaccharides from Escherichia coli O111:B4 were obtained from Sigma-Aldrich (St. Louis, MO, USA).

### 2.2. Extraction

A mixer (KWG-150, Sunway Electric Manufacture [He Shan] Co., Ltd., China) was used for homogenization. First, 5 g of sample with 100 mL of 80% ethanol was homogenized and subsequently incubated in shaking incubator for 1 d (DH. WIS 02011, DAEHAN Scientific Co., Ltd. Korea). Thereafter, the homogenized sample was centrifuged for 10 min at 15,000 rpm (Mega21R, Hanil, Korea) and the supernatant was collected. The supernatant was then filtered through a Minisart syringe filter (0.45 μm pore size) and concentrated using a rotary evaporator (N-1000, Eyela, Japan) under reduced pressure until the sample volume reached 20 mL. The final sample was stored at −80 °C until quantitative analysis.

### 2.3. Color Analysis

The color of the pink pepper samples was determined using a colorimeter (NE-6000, Nippon Denshoku, Tokyo, Japan). The color of each sample was represented as L*, a*, and b* measurements. L* represents lightness (L* = 0, black; and L* = 100, white), a* represents redness, and b* represents yellowness. For accurate analysis, all five replicates were performed according to the method described by Yang et al. [16].

### 2.4. Analysis of Sugar Composition

Quantification of the sugar composition was performed as reported by Kim and Shin [17]. Sample extracts were diluted 20-fold in distilled water and then filtered through a 0.45 μm syringe filter. Instrumental analyses were performed using an UltiMate 3000 high-performance liquid chromatography (HPLC) system (Thermo Fisher Scientific, MA, USA) with a Refractomax 520 refractive index detector (ERC Inc., Saitama, Japan). A high-performance carbohydrate column (250 × 4.6 mm i.d., 4 μm, Waters, USA) was used at 30 °C for identification of individual sugars. The mobile phase was 75% acetonitrile in distilled water and the flow rate was 1.0 mL/min; 10 μL of each sample was injected. We utilized 100, 250, 500, 750, and 1000 mg/100 g of fructose and glucose as reference materials to produce standard calibration curves. Quantification was performed three times and the results were expressed as mg/100 g.

### 2.5. Total Flavonoids and Total Phenolics

The amount of total flavonoids in the extracts was measured using a colorimetric assay as described by Meyers et al. [18]. First, 1 mL of sample extract was diluted five-fold with distilled water along with 0.3 mL of 5% sodium nitrite in a 15 mL tube. The solution was reacted at room temperature (RT) for 5 min. Subsequently, 0.3 mL of 10% aluminum chloride was added and mixed thoroughly. The reagents were reacted at RT for 6 min. After that, 2 mL of 1N sodium hydroxide solution was added and the final volume of the sample solution was adjusted to 10 mL with distilled water. Finally, the absorbance of the solution was measured at a wavelength of 510 nm using a spectrophotometer (Optizen POP, Mecasys, Korea). The results were expressed as mg catechin equivalents (CE)/100 g.

Total phenolic content was determined using the colorimetric method as described by Meyers et al. [18]. First, 0.2 mL of sample extract, 2.6 mL of distilled water, and 0.2 mL of Folin–Ciocalteu reagent were mixed in a 15 mL tube. The solution was reacted at RT for 6 min and then 2 mL of 7% sodium carbonate was added. Next, the solution was reacted for 90 min at RT in a darkroom. Finally, the absorbance of the reaction mixture was measured at 750 nm using a spectrophotometer (Optizen POP). We utilized gallic acid as a reference material to produce a standard curve. Total phenolic content was expressed as mg gallic acid equivalents (GAE)/100 g.

### 2.6. Polyphenol Quantification

The polyphenol content of the extracts was determined using a modified method [16]. The extracts were diluted 20 fold (KH_2_PO_4_:MeOH:Water = 2:3:15) and subsequently filtered through a 0.45-µm syringe filter. After that, samples were analyzed by HPLC (Thermo Fisher UltiMate 3000, Thermo Fisher Scientific, Germany). The analytical column was an Eclipse XDB C-18 (150 × 4.6 mm, 5 μm, Agilent, USA) at 40 °C. The mobile phase was 3% acetic acid with a flow rate of 1.0 mL/min; 10 μL of each sample was injected. Detection was performed using a photo diode array detector set to a wavelength of 280 nm. Five individual reference materials were utilized to produce standard calibration curves: piperine, gallic acid, protocatechuic acid, epicatechin, and p-coumaric acid. Polyphenol content was expressed as mg/100 g.

### 2.7. Radical Scavenging Effect

The 2,2-diphenyl-1-picrylhydrazyl (DPPH) radical scavenging activity of the extracts was determined using a previously described method with modifications [19]. First, 0.1 mM DPPH solution was prepared and diluted with 80% methanol to an absorbance of 0.65 ± 0.02 at a wavelength of 517 nm. After that, 50 μL sample extract was diluted 60-fold with DPPH solution and reacted at RT for 30 min. Finally, the absorbance of the sample solution was measured at a wavelength of 517 nm using a spectrophotometer (Optizen POP, Mecasys, Korea). Radical scavenging activity was expressed as mg vitamin C equivalents (VCE)/100 g.

The 3-ethylbenzothiazoline-6-sulphonic acid (ABTS) radical scavenging activity of the extracts was estimated using a modified version of a previous ABTS radical method [19]. First, 1 mM 2,2′-azobis (2-amidinopropane) dihydrochloride (AAPH) and 2.5 mM ABTS were completely dissolved and mixed in phosphate buffered saline (PBS). The sample solution was reacted for 40 min at 70 °C in a water bath, then immediately cooled from RT. The working ABTS solution was diluted with PBS to an OD_734_ of 0.63 to 0.67. Thereafter, 20 μL of the sample was diluted 50-fold with working ABTS solution and reacted at 37 °C for 10 min. The absorbance of the solution was measured at 734 nm using a spectrophotometer (Optizen POP, Mecasys, Korea) and the results were expressed as mg VCE/100 g.

### 2.8. Cell Culture

RAW264.7 cells were purchased from the Korean Cell Line Research Foundation at the Korean Cell Line Bank (Seoul, Korea) and were maintained in Dulbecco’s modified Eagle’s medium containing 10% fetal bovine serum and 1% penicillin and streptomycin. RAW264.7 cells were passaged at 70–80% confluence and cultured in an incubator at 37 °C with 5% CO_2_ (Thermo Fisher Scientific, MA, USA).

### 2.9. Cell Viability

RAW264.7 cells were seeded at 2 × 10^5^ cells/mL on a 96-well plate and cultured overnight in an incubator at 37 °C with 5% CO_2_. The medium was replaced with medium containing PPB and PPS for 24 h and then 10 μL of 5 mg/mL MTT solution (Sigma-Aldrich) was added to each well. After incubation for 4 h, the supernatant was discarded and then cells were dissolved with 100 μL of dimethyl superoxide (Sigma-Aldrich). Cell viability was measured with a microplate reader (Bio-Rad Inc., Hercules, CA, USA) at 595 nm.

### 2.10. Measurement of Intracellular ROS

Intracellular ROS were measured using 2′,7′-dichlorofluorescein diacetate (DCF-DA) with a fluorescence reader and fluorescence microscopy. RAW264.7 cells were seeded at 1 × 10^5^ cells/mL in a 96-well plate and cultured overnight. Cells were pre-treated with PPB and PPS and then stimulated with LPS for 24 h. After washing with 200 μL PBS, cells were incubated with 20 μM DCF-DA in serum-free media for 30 min. The dye solution was discarded and the cells were washed twice. ROS production was measured at an excitation wavelength of 485 nm and an emission wavelength of 530 nm using a fluorescence reader (Molecular Devices, CA, USA). Cellular ROS were measured by fluorescence microscopy (Leica, Wetzlar, Germany) using LAS X microscope software (Leica).

### 2.11. Nitrite Assay

RAW264.7 cells were seeded at 2 × 10^5^ cells/mL in a 96-well plate and cultured overnight in an incubator at 37 °C with 5% CO_2_. Nitrite production was measured with Griess reagents. Cells were pre-treated with pink pepper extracts (PPE) for 1 h before 1 μg/mL LPS treatment for 24 h. The culture supernatant was transferred to a 96-well plate and reacted with equal volumes of Griess reagent (0.2% N-[1-naphthyl]-ethylenediamine dihydrochloride and 1% sulfanilamide in 5% phosphoric acid). After 30 min, absorbance was measured 550 nm using a microplate reader (Bio-Rad Inc.).

### 2.12. Western Blot Analysis

RAW264.7 and HaCaT cells were seeded at 3 × 10^5^ cells/mL in 100 mm dishes and cultured for 24 h. Cells were pre-treated with PPE at different concentrations (50 and 100 μg/mL) for 1 h and then treated with LPS (1 μg/mL). Cells were washed twice in ice-cold PBS, scraped with cell lysis buffer (Cell Signaling Technologies) containing protease and phosphatase inhibitor (Thermo Fisher Scientific), and then collected. After 30 min, collected cells were centrifuged at 13,000 rpm for 15 min. Subsequently, transferred supernatants were stored until use for Western blot assay. For protein quantification, cell lysate was determined with reference to a standard curve of bovine serum albumin. A DC protein assay kit (Bio-Rad Inc.) was used to measure 30 μg of lysate protein. Proteins were separated by electrophoresis on 10% sodium dodecyl sulfate–polyacrylamide gel (SDS–PAGE) and then transferred to a polyvinylidene difluoride (PVDF) membrane (Millipore, State of New Jersey, MA, USA). Transferred proteins were blocked with tris-buffered saline with Tween 20 containing 5% skim milk (BD Biosciences, San Jose, CA, USA) for 1 h at RT. After blocking, each membrane was incubated with specific primary antibodies overnight at 4 °C. After washing three times with PBS, the proteins were incubated with horseradish peroxidase–conjugated secondary antibodies (Thermo Fisher Scientific) and primary antibodies for 1 h at RT. Protein signals were detected using a chemiluminescence substrate (ATTO, Tokyo, Japan). Then, GeneGnome XRQ (Syngene, Cambridge, UK) was used for protein visualization.

### 2.13. Statistical Analysis

For multiple comparison, the SAS 9.4 statistical program (SAS Institute Inc., Cary, NC, USA) was used to perform one-way analysis of variance and Duncan’s multiple range test. These analyses were used to determine the statistical significance of each average value (*p* < 0.05). Experimental results are expressed as mean ± standard deviation from triplicate determination.

## 3. Results and Discussion

### 3.1. Color and Sugar Content Analysis

**Figure 1:**
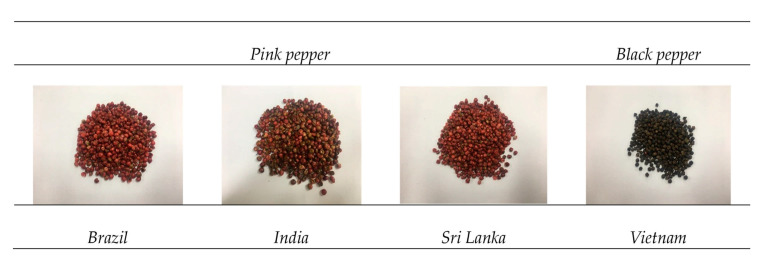
Photographs of dried pink peppers from different countries and black pepper.

Although *S. molle* is a known as American pepper, it has distinctive color and chemical compositions compared to black pepper. While the chemical and physiological properties of black pepper have been well studied [20,21], the chemical composition and physiological activity of *S. molle* are mainly unknown. Therefore, we compared the in vitro antioxidant effect of pink peppers from three different countries and black pepper as well as analyzed the chemical compositions of *S. molle* fruits. To the best of our knowledge, this is the first report of this approach and these findings. Pink and black peppers have distinctive colors and shapes. Pictures of the dried pink peppers from three countries and the BPV used in this experiment are shown in Figure 1. 

To compare the general characteristics of pink and black peppers, we analyzed the color and carbohydrate contents of the peppers. Table 1 summarizes the Hunter L, a, b values and fructose and glucose contents of PPB, PPI, and PPS. We determined fructose, glucose, sucrose, and maltose content in the pink and black peppers (S1) using HPLC assay [16]. While fructose and glucose were not detected in BPV, the pink peppers showed relatively high levels of both monosaccharides. PPS and PPB had the highest fructose content (11,829.82 ± 23.73 mg/100 g) and glucose content (9816.07 ± 36.51 mg/100 g). In the HPLC chromatogram of sugar analysis, we detected two peaks that were consistent with the fructose and glucose standards (Figure S1). Feriani et al. identified fucose (10.90% ± 0.024%) in *S. molle* fruits from Tunisia, and interestingly, glucose was not detected [15]. On the HPLC chromatogram obtained for pink pepper, we confirmed two peaks that were consistent with the standard peaks of fructose and glucose, but no peaks corresponding to fucose were detected (Figure S1). Therefore, we ruled out the hypothesis that fucose would be present in considerable amount in commercial *S. molle*. Further, Solis et al. reported that fresh and residue seeds of *S. molle* contained 16% glucose; however, they did not confirm fructose and fucose [22]. In our method, we used 70% EtOH for sample extraction, while Feriani et al. used hot water extraction for *S. molle* fruits from Tunisia. Therefore, these differences in monosaccharide contents seem to be due to the differences in the origin of the peppers, cultivation environment, and the extraction method. 

### 3.2. Total Flavonoid Contents and Total Phenolic Contents

Several studies have characterized the chemical and physiological functions of the essential oil [13,14], carbohydrates [15], and fragrance ingredients [12] of *S. molle*. The essential oil and fragrance ingredients are useful for food seasoning. To obtain essential oil and carbohydrates from the pink peppers, the researchers used water as an extraction solvent [12]. Because there are few studies on the application of *S. molle* fruits as nutraceuticals, we analyzed the phenolic and flavonoid content and antioxidant capacity of *S. molle* and evaluated its potential as a nutraceutical ingredient and antioxidant.

Total phenolic contents were expressed in GAE per 100 g. The highest total phenolic and flavonoid contents were found in PPB (1607.80 ± 21.11 mg GAE/100 g) and BPV (344.24 ± 3.78 mg CE/100 g), respectively (Table 2). Interestingly, PPB, PPI, and PPS had higher total phenolic and lower total flavonoid contents than BPV. In our previous study, we determined the total flavonoid and total phenolic contents of green peppers to be 1083.43 ± 8.24 mg CE/100 g and 1414.63 ± 10.56 mg CE/100 g, respectively [23]. Even though direct comparison of total flavonoid and total phenolic contents among green and pink peppers is limited, pink pepper may have higher total flavonoid and lower total phenolic than green and black peppers.

### 3.3. Quantification of Major Compounds in Peppers by HPLC

Piperine, a phenolic component, is a representative component of peppers and is responsible for the pungency and flavor of black pepper [24]. Three repeated quantitative analyses of PPB, PPI, and PPS showed piperine concentrations of 134.6 mg/100 g, 101.1 mg/100 g, and 120.67 mg/100 g, respectively (Table 3). Meanwhile, piperine concentrations in BPV were determined to be 4097.53 mg/100 g (Table 4). Although PPB, PPI, and PPS have higher DPPH and ABTS radical scavenging capacities, their piperine concentrations were lower than that of BPV (Table 3 and Table 4). Giuffrida et al., identified a volatile compound and 10 carotenoids in the essential oil of *S. molle* [12]. Feuerisen et al. confirmed that anthocyanins, bioflavonoids, and gallotannins are present in *S. terebinthifolia* and *S. molle* by using UHPLC-MS/MS analysis [25]. In the present study, we further determined the polyphenol contents of pink peppers and compared them to those of black pepper. The results of three repeated quantitative analyses of PPB, PPI, and PPS polyphenols are listed in Table 4. The most abundant polyphenol in the pink peppers was gallic acid; its content was highest in PPI (657.59 ± 5.25 mg/100 g) and lowest in PPS (168.15 mg/100 g) (Table 4). Additionally, gallic acid, protocatechuic acid, epicatechin, and p-coumaric acid were detected in the pink peppers, but not in the black pepper. Therefore, the differences in radical scavenging capacity between pink peppers and black pepper may originate in the different chemical compositions of the peppers.

Parra et al. quantified the phenolic compounds such as gallic acid (43.60 mg/100 g DW), protocatechuic acid (61.99 mg/100 g DW), and *p*-Coumaric acid (9.32 mg/100 g DW) in *Origanum vulgare* L. by using UHPLC-DAD [26]. Zhang et al. identified phenolic contents including gallic acid (11.30 ± 0.28 μg/g) and protocatechuic acid (39.53 ± 0.80 μg/g) from *Lycium ruthenicum* Murray by UPLC-Q-Orbitrap MS [27]. These extracts have also shown strong antioxidant activities, and the authors indicated the relevance of high phenolic compounds. However, it was not possible to directly compare whether the content of polyphenols in pink pepper contributes to the antioxidant activity as there have been no previous studies. Therefore, further research is needed for the bioactivity evaluation of pink pepper.

### 3.4. Radical Scavenging Effects

Phenolic compounds and flavonoids contribute to the antioxidant capacities of natural materials [28]. Based on the high phenolic and flavonoid contents of the peppers, we further evaluated their ability to scavenge DPPH and ABTS radicals. The radical scavenging capacities of DPPH and ABTS were measured to determine antioxidant activity and expressed as VCE. The greatest DPPH and ABTS radical scavenging capacity were found in PPI (4081.92 ± 34.39 mg VCE/100 g and 2845.12 ± 3.91 mg VCE/100 g) (Table 3). The results of a recent study showed that *S. mole* polysaccharide has significant DPPH and ABTS radical scavenging effects [15]. In our previous study, the range of DPPH radical scavenging capacity of the green and black peppers was found to be from 522.83 to 194.42 mg VCE/100 g, and the range of ABTS radical scavenging capacity was 1941.91 to 526.45 mg VCE/100 g [23]. Therefore, we suggest that the radical scavenging capacity of pink pepper is higher than that of black and green pepper.

### 3.5. Pink Peppers Inhibited LPS-Induced ROS Production and Increased HO-1 Expression in RAW264.7 Cells

**Figure 2:**
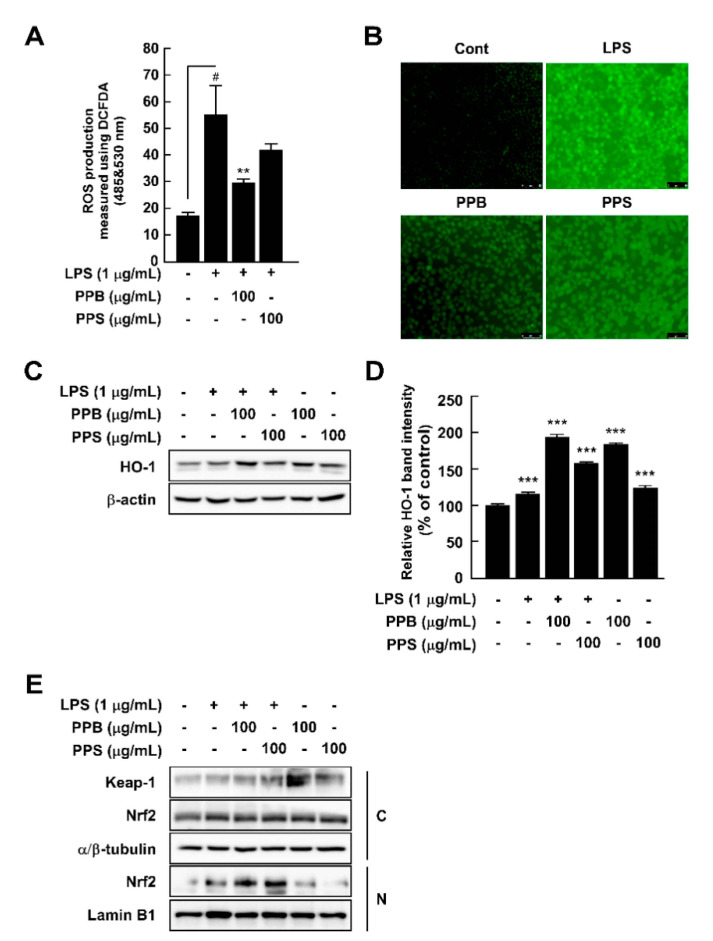
Effects of pink pepper extract (PPE) on lipopolysaccharides (LPS)-induced reactive oxygen species (ROS) production and HO-1 expression in RAW264.7 cells. (**A**) PPE suppressed LPS-induced ROS production in RAW264.7 cells. (**B**) Pink peppers from Brazil (PPB) suppressed LPS-induced ROS production at 100 μg/mL. Images show representative micrographs of cells under fluorescence microscope. (**C**) PPB and Pink peppers from Sri Lanka (PPS) significantly elevated HO-1 expression independently of LPS presence in RAW264.7 cells. (**D**) Quantification of HO-1 expression. (**E**) PPB and PPS enhanced Nrf2 translocation from the cytosol to the nucleus. Expression levels of HO-1, Keap-1, and Nrf2 were determined by Western blot. Values represent the mean ± standard deviation of three independent experiments. ^#^ *p* < 0.05 between control versus LPS-exposed cells (no PPE); ** *p* < 0.01. *** *p* < 0.001 represents a significant difference compared with the control group.

Overproduction of ROS damages cells and host organs and subsequently causes inflammation and diseases [29]. Although PPB and PPI have higher DPPH and ABTS radical scavenging capacities and phenolic compound contents than those of PPS, to evaluate whether this effect is related to the antioxidant and anti-inflammatory effects in cells, we selected PPB and PPS as having the highest and lowest effects. By using DCFH-DA as a chemical probe for ROS, we determined that PPB significantly suppressed LPS-induced ROS production in RAW264.7 cells (Figure 2A,B). Increased ROS levels are affected by various factors, such as antioxidant enzymes. Among many antioxidant enzymes, HO-1 has been reported to correlate with increased intracellular ROS production [30]. In the present study, PPB and PPS significantly induced HO-1 expression in RAW264.7 cells independently of LPS (Figure 2C,D). Additionally, PPB and PPS increased nuclear Nrf2 translocation (Figure 2E). The results of several studies have suggested the DPPH and ABTS radical scavenging capacity of *S. molle* [14,15,31], but the effect of this pepper on cellular antioxidant and antioxidative enzyme expression is unclear. Here, we found that the antioxidant capacity of *S. molle* fruit is due to an increase in Nrf2 translocation from the cytosol to the nucleus and HO-1 expression in RAW264.7 cells. Additionally, we confirmed that the radical scavenging capacity at the test-tube level and the phenolic compound content highly correlated with the antioxidant activity in RAW264.7 cells. 

### 3.6. Effects of PPE on LPS-Induced Nitrite Production, iNOS and COX-2 Expression, and p65 and MAPK Phosphorylation in RAW264.7 Cells

**Figure 3:**
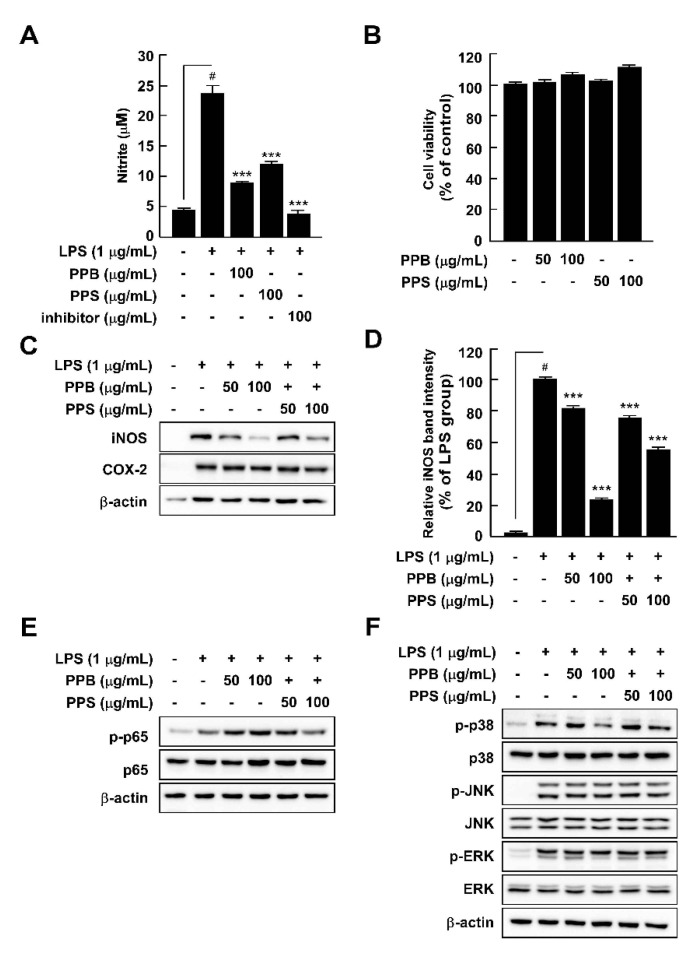
Effects of pink pepper extract (PPE) on lipopolysaccharides (LPS)-mediated nitrite production, cell viability, iNOS and COX-2 expression, and p65 and MAPK phosphorylation in RAW264.7 cells. (**A**) PPE suppressed LPS-induced nitrite production in RAW264.7 cells. (**B**) PPE did not affect cell viability for 24 h. (**C**) PPE inhibited LPS-induced iNOS expression in RAW264.7 cells, but did not alter COX-2 protein expression. (**D**) Quantification of iNOS expression. (**E**) PPE suppressed LPS-induced phosphorylation of p65 in RAW264.7 cells. (**E**) PPE did not affect LPS-induced phosphorylation of p65 in RAW264.7 cells (**F**) PPE inhibited LPS-induced phosphorylation of p38, but not JNK and ERK. Data are presented as the mean ± standard deviation of three independent experiments. ^#^ *p* < 0.05 between control and LPS-exposed cells (no PPE); *** *p* < 0.001.

Abnormal NO production is a vital marker of inflammation in response to LPS treatment [32]. PPB and PPS significantly suppressed LPS-induced nitrite production without cell toxicity in comparison to pre-treatment with an NF-κB inhibitor, parthenolide (Figure 3A,B). Because NO is mainly produced by iNOS, we further explored the effect of PPB and PPS on LPS-mediated iNOS expression in RAW264.7 cells. PPB and PPS significantly inhibited iNOS expression, but not COX-2 expression (Figure 3C). Because the association between iNOS expression and upstream regulatory signaling pathways such as the NF-κB and MAPK signaling pathways is well known, we subsequently assessed the effect of PPB and PPS on the LPS-induced NF-κB and MAPK signaling pathways in RAW264.7 cells. PPE inhibited LPS-induced phosphorylation of p38, but not p65, JNK, or ERK (Figure 3E,F). Gu et al. suggested that phenolic and volatile extracts of six berries suppressed LPS-mediated abnormal NO production by 48–94% [33]. In the present study, the inhibitory effect of PPB and PPS on LPS-induced NO production was 86.3% and 67.7%, respectively (Figure 3A). Therefore, it seems that pink pepper exhibits inhibitory activity similar to that of berries, which are well known for their inhibition of NO production [33]. Interestingly, although materials that have a suppressive effect on LPS-induced NO production mainly regulated phosphorylation of p65 [23,33,34], a catalytic subunit of NF-κB, PPB and PPS only affected the phosphorylation of p38 in MAPKs in RAW264.7 cells. Our previous study also reported that ultrasonicated seaweed extract only affected phosphorylation of MAPKs and activator protein (AP-1), and not NF-κB [7]. Although further study is required to determine whether PPB and PPS regulate LPS-induced activator protein-1 activity, p38 is a major target of PPB and PPS in iNOS expression and NO production.

### 3.7. Effects of PPE on UVB-Induced COX-2 Expression and MAPK Phosphorylation in HaCaT Cells

**Figure 4:**
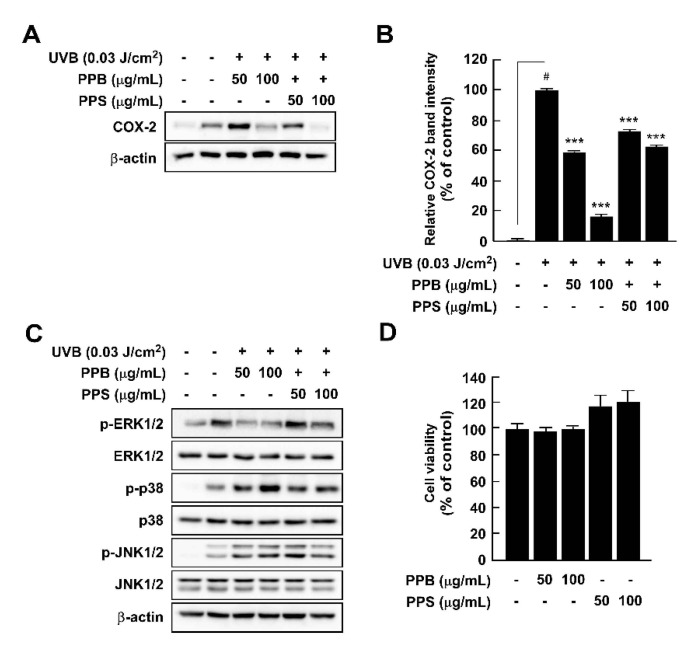
Effects of pink pepper extract (PPE) on UVB-induced COX-2 expression and MAPK phosphorylation in HaCaT cells. (**A**) PPE suppressed UVB-induced COX-2 expression in HaCaT cells. Cells were treated with the indicated concentration of PPE for 1 h and then stimulated with UVB (0.03 J/cm^3^) for 4 h. (**B**) Quantification of COX-2 expression by PPB and PPS. (**C**) PPE suppressed UVB-induced phosphorylation of ERK1/2, but not p38 and JNK1/2. Cells were treated with PPE for 1 h and then stimulated with UVB (0.03 J/cm^3^) for 30 min. (**D**) PPB and PPS did not affect the cell viability at the tested concentrations. The cells were treated with increasing concentration of PPB and PPS for 24 h. Phosphorylated MAPK and COX-2 expression were detected by Western blot. Data are presented as mean ± standard deviation of three independent experiments. ^#^ *p* < 0.05 between control and LPS-exposed cells (no PPE); *** *p* < 0.001.

Although UV irradiation of human skin has positive effects, such as killing pathogens and aiding synthesis of vitamin D, acute and chronic exposure to UV light results in skin inflammation and skin cancer, respectively [9,35]. We investigated whether pink peppers suppressed UVB-induced COX-2 expression and phosphorylation of MAPKs, which are major regulators of *cox-2* gene expression. Our results showed that PPB and PPB significantly suppressed UVB-induced COX-2 expression in HaCaT cells (Figure 4A,B). We previously suggested that botanical extracts and compounds could act as chemopreventive agents via suppression of UVB-induced COX-2 expression in vitro and in vivo [8,9,35,36,37]. Because PPB and PPS have significant effects on UVB-induced COX-2 expression, they could be candidates for chemopreventive materials. MAPKs are key regulators of COX-2 expression as they regulate activator protein-1 activity [38]. Among the MAPKs, PPB and PPS affected phosphorylation of ERK, but not p38 or JNK1/2 in HaCaT (Figure 4C). We suggest that it is due to the difference in phenolic contents by demonstrating COX-2 expression and MAPK phosphorylation activities, which are higher in PPB than in PPS, similar to antioxidant activity.

## 4. Conclusions

Here, we confirmed that PPB, PPI, and PPS have greater radical scavenging capacities, higher fructose and glucose contents, and higher total phenolic contents than BPV. Additionally, we confirmed that pink pepper and black pepper have different chemical compositions. BPV is higher in piperine; meanwhile, gallic acid, protocatechuic acid, epicatechin, and p-coumaric acid were detected only in PPB, PPI, and PPS. Furthermore, PPB significantly suppressed LPS-induced ROS production with an increase in HO-1 expression in RAW264.7 cells. PPB and PPS exerted anti-inflammatory effect on LPS-induced iNOS expression and UVB-induced COX-2 expression via regulation of p38 and ERK1/2 phosphorylation in RAW264.7 cells and HaCaT cells, respectively. Collectively, the findings of our study indicate that pink pepper is a promising nutraceutical with superior antioxidant and anti-inflammatory properties. 

## Figures and Tables

**Table 1:** Color and sugar composition of pink and black peppers.

	Region	L	a	b	Fructose (mg/100 g)	Glucose (mg/100 g)
Pink	Brazil	28.87 ± 0.01 ^b^	11.94 ± 0.01 ^a^	11.41 ± 0.004 ^b^	11,507.21 ± 90.5 ^b^	9816.07 ± 36.51 ^a^
	India	25.02 ± 0.09 ^d^	6.71 ± 0.05 ^b^	8.55 ± 0.04 ^d^	9528.74 ± 46.67 ^c^	6181.37 ± 315.61 ^b^
	Sri Lanka	27.36 ± 0.03 ^c^	11.92 ± 0.06 ^a^	10.89 ± 0.05 ^c^	11,829.82 ± 23.73 ^a^	9758.15 ± 330.28 ^a^
Black	Vietnam	43.65 ± 0.01 ^a^	3.81 ± 0.02 ^c^	13.16 ± 0.01 ^a^	N.D.	N.D.

^(1)^ Values are the average of experiments (*n* = 5) and represented as mean ± standard deviation; different letters (a–d) in a column indicate values that are significantly different at *p* < 0.05.

**Table 2:** Total flavonoid and total phenolic contents of pink and black peppers.

	Region	Total Phenolic Content (mg GAE/100 g)	Total Flavonoid Content (mg CE/100 g)
Pink	Brazil	1607.80 ± 21.11 ^a^	266.67 ± 2.42 ^b^
	India	1588.29 ± 17.88 ^a^	230.30 ± 2.10 ^d^
	Sri Lanka	1250.08 ± 10.75 ^b^	248.89 ± 5.60 ^c^
Black	Vietnam	794.47 ± 17.19 ^c^	344.24 ± 3.78

^(1)^ Values are the average of experiments (*n* = 3) and represented as mean ± standard deviation. CE, catechin equivalents; GAE, gallic acid equivalents; different letters (a–d) in a column indicate values that are significantly different at *p* < 0.05.

**Table 3:** Radical scavenging capacities of pink and black peppers.

	Region	DPPH (mg VCE/100 g)	ABTS (mg VCE/100 g)
Pink	Brazil	4015.32 ± 13.00 ^a^	2741.25 ± 19.69 ^a^
	India	4081.92 ± 34.39 ^a^	2845.12 ± 3.91 ^a^
	Sri Lanka	2812.30 ± 10.81 ^b^	1956.96 ± 54.26 ^b^
Black	Vietnam	271.45 ± 12.01	861.92 ± 83.23

^(1)^ Values are the average of experiments (*n* = 3) and represented as mean ± standard deviation. VCE, vitamin C equivalents; DPPH, 2,2-diphenyl-1-picrylhydrazyl; ABTS, 3-ethylbenzothiazoline-6-sulphonic acid; different letters (a–d) in a column indicate values that are significantly different at *p* < 0.05.

**Table 4:** Individual polyphenol profiles of pink and black peppers.

	Region	Piperine (mg/100 g)	Gallic Acid (mg/100 g)	Protocatechuic Acid (mg/100 g)	Epicatechin (mg/100 g)	*p*-Coumaric Acid (mg/100 g)
Pink	Brazil	134.60 ± 3.20 ^b^	526.72 ± 6.06 ^b^	144.85 ± 0.71 ^b^	85.91 ± 2.88 ^a^	115.92 ± 5.00 ^b^
	India	101.10 ± 2.84 ^c^	657.59 ± 5.25 ^a^	237.52 ± 0.64 ^a^	89.24 ± 2.04 ^a^	151.33 ± 7.07 ^a^
	Sri Lanka	120.67 ± 1.91 ^bc^	168.15 ± 1.43 ^c^	29.47 ± 0.18 ^c^	38.26 ± 1.28 ^b^	48.24 ± 1.28 ^c^
Black	Vietnam	4097.53 ± 46.87 ^a^	N.D	N.D	N.D	N.D

^(1)^ Values are the average of experiments (*n* = 3) and represented as mean ± standard deviation. N.D, not detected; different letters (a–d) in a column indicate values that are significantly different at *p* < 0.05.

## Data Availability

Data is contained within the article and supplementary material.

## Supplementary Materials

The following are available online at https://www.mdpi.com/article/10.3390/antiox10071062/s1, Figure S1: Chromatograms for fructose, glucose, sucrose, and maltose in the pink and black peppers.
