# Supplementary material for: Analysis of the Chemical, Antioxidant, and Anti-Inflammatory Properties of Pink Pepper (Schinus molle L.)
Source: Antioxidants (Basel). 2021 Jun 30;10(7):1062. doi: 10.3390/antiox10071062 (PMC8300677; doi:10.3390/antiox10071062)
Supplement: Supplementary file 1 [file antioxidants-10-01062-s001.zip › antioxidants-1282498-supplementary.pdf]

Supplementary Figure S1. Chromatograms for fructose, glucose, sucrose, and maltose in the pink and black peppers

Samples	Chromatogram
**Blank**	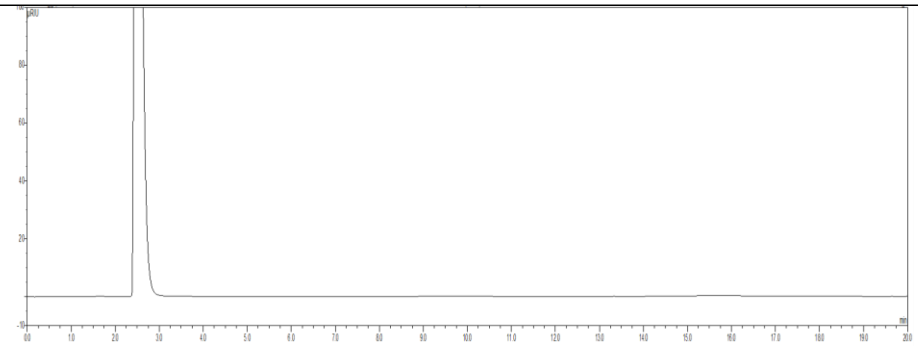
**Fucose 1%**	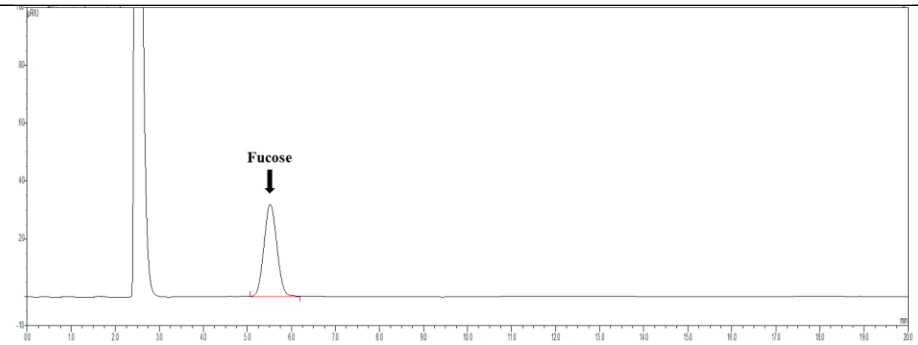
**Sugar 4mix 1%<br>(Fructose, Glucose,<br>Sucrose, Maltose)**	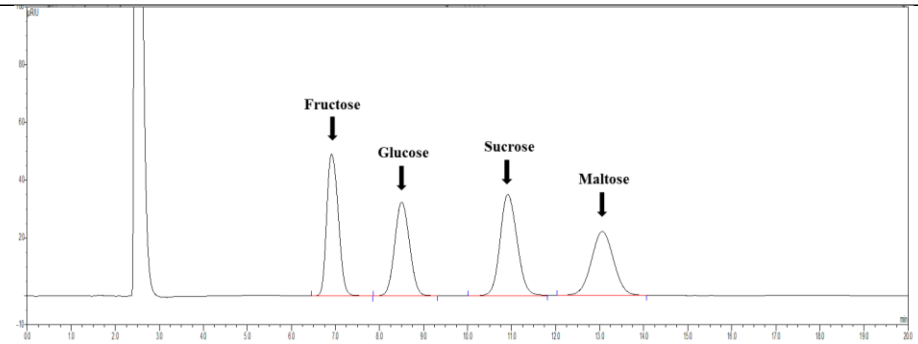
**Pink pepper,<br>Brazil**	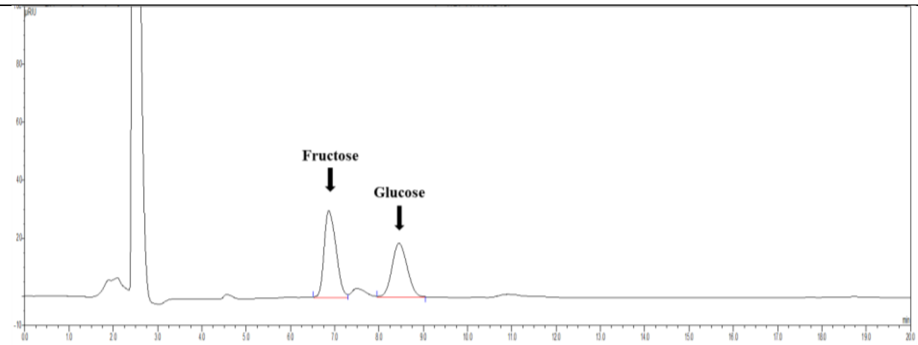
**Pink pepper,<br>India**	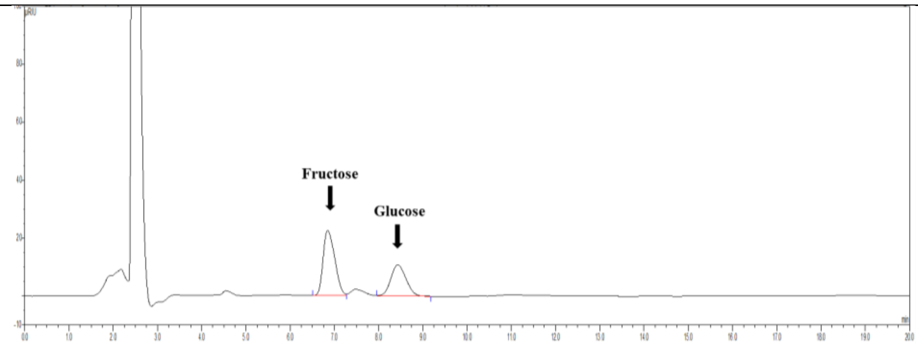

**Pink pepper, Sri Lanka**	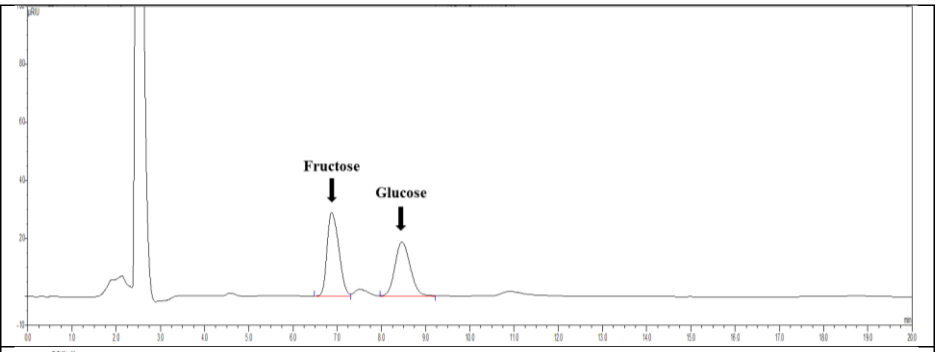
**Black pepper, Vietnam**	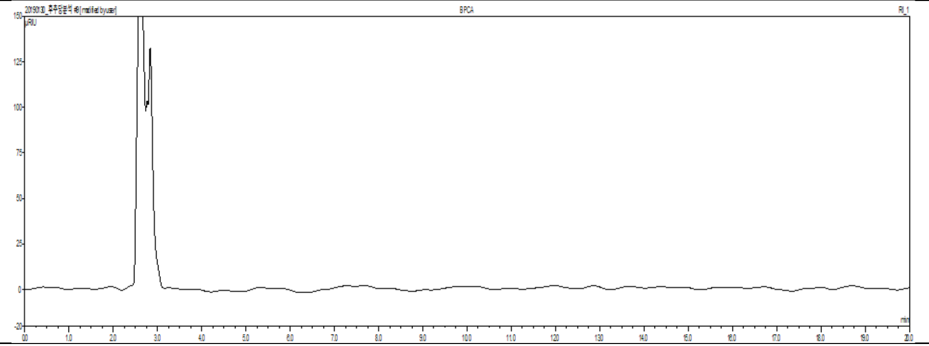
